# S100P interacts with integrin α7 and increases cancer cell migration and invasion in lung cancer

**DOI:** 10.18632/oncotarget.4987

**Published:** 2015-07-21

**Authors:** Ya-Ling Hsu, Jen-Yu Hung, Yung-Yu Liang, Yi-Shiuan Lin, Ming-Ju Tsai, Shah-Hwa Chou, Chi-Yu Lu, Po-Lin Kuo

**Affiliations:** ^1^ Graduate Institute of Medicine, College of Medicine, Kaohsiung Medical University, Kaohsiung, Taiwan; ^2^ Division of Pulmonary and Critical Care Medicine, Kaohsiung Medical University Hospital, Kaohsiung, Taiwan; ^3^ School of Medicine, College of Medicine, Kaohsiung Medical University, Kaohsiung, Taiwan; ^4^ Division of Chest Surgery, Department of Surgery, Kaohsiung Medical University Hospital, Kaohsiung, Taiwan; ^5^ Department of Biochemistry, Kaohsiung Medical University, Kaohsiung, Taiwan; ^6^ Institute of Clinical Medicine, College of Medicine, Kaohsiung Medical University, Kaohsiung, Taiwan; ^7^ Institute of Medical Science and Technology, National Sun Yat-Sen University, Kaohsiung, Taiwan

**Keywords:** S100P, ZEB1, FAK, metastasis, oncogene

## Abstract

S100P, a Ca2^+^ binding protein, has been shown to be overexpressed in various cancers. However, its functional character in lung cancer remains largely unknown. In this study, we show that S100P increases cancer migration, invasion and metastasis in lung cancer cells. Ectopic expression of S100P increases migration, invasion and EMT in less invasive CL1-0 lung cancer cells. Conversely, knockdown of S100P suppressed migration and invasion, and caused a reversion of EMT in highly invasive lung cancer cells. These effects were transduced by increasing the interaction of S100P with integrin α7, which activated focal adhesion kinase (FAK) and AKT. Blocking FAK significantly decreased S100P-induced migration by decreasing Src and AKT activation, whereas inhibiting AKT reduced S100P upregulation on ZEB1 expression. Further study has indicated that S100P knockdown prevents the spread of highly metastatic human lung cancer in animal models. This study therefore suggests that S100P represents a critical activator of lung cancer metastasis. Detection and targeted treatment of S100P-expressing cancer is an attractive therapeutic strategy in treating lung cancer.

## INTRODUCTION

Lung cancer is the one of the most commonly diagnosed malignancies, with a high mortality rate worldwide. Non-small-cell lung cancer (NSCLC) accounts for approximately 85% of all lung cancers [1]. Surgical intervention is the major treatment for resectable tumors, while chemotherapy or radiotherapy are standard forms of care for unresectable, advanced NSCLC. Unfortunately, the overall five year survival rate of advanced NSCLC is still < 15% [2, 3], with metastasis as the major cause of treatment failure. Thus, understanding the metastatic mechanism and establishing novel therapeutic targets and strategies is of great importance in targeting lung cancer.

S100P, a member of the S100 family of small EF-hand calcium-binding proteins, exhibits different cell biologic functions extra- and intra-cellularly by direct targeting with specific proteins. Its expression is upregulated in some cancers, including colon, pancreatic and endometrial cancers, and it is associated with poor clinical outcomes and decreased chemosensitivity [4-6]. Receptor for advanced glycation end products (RAGE), a major extracellular binding target of S100P, has been associated with S100P-mediated cancer progression by triggering the oncogenic signaling pathway microRNA-155 and NF-κB in colon and pancreatic cancers, respectively [7, 8]. S100P has also been reported to directly interact with cell motion related protein nonmuscle myosin II and EZRIN, resulting in increased cell migration [9, 10]. However, the exact role of S100P in the invasive capacity of human lung cancer remains unknown.

Integrins, large and complex transmembrane glycoproteins belonging to adhesion receptors, modulate various cell functions upon ligand binding [11]. Focal adhesion kinase (FAK) is the most important mediator of integrin, regulating cell motility, proliferation, and cellular stress response to ionizing radiation and chemotherapy [12, 13]. One member of the S100 family protein, S100A7, has been shown to interact with the integrin β6 subunit. This interaction activates integrin ανβ6, resulting in increased cell invasion [14]. Other S100 proteins may likewise interact with and regulate different integrin isoforms to cause an overall enhancement of cancer progression. However, how such enhancement occurs remains unknown.

The present study demonstrates that S100P binds preferentially to integrin α7 with the activation of FAK and AKT signaling. Consequently, the metastatic characteristics of lung cancer cells are enhanced. Thus, inhibition of S100P may provide a novel therapeutic target for combating lung cancer metastasis.

## RESULTS

### Elevated S100P expression in highly invasive lung cancer cells and tumor regions

**Figure 1:**
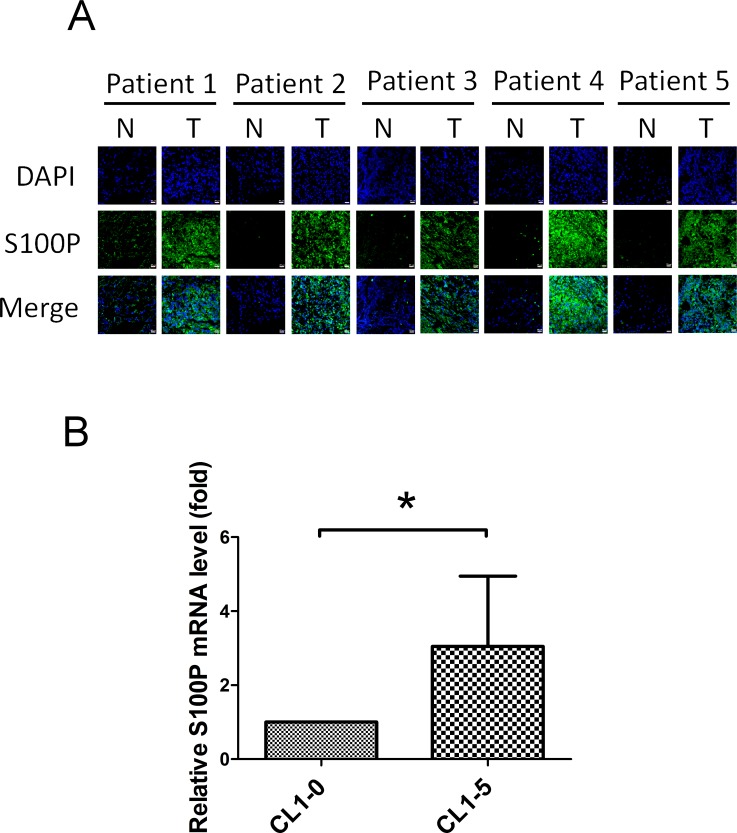
Elevated S100P expression in highly invasive lung cancer cells and tumor regions **A.** The S100P level in nontumorous and tumorous regions of lung cancer patients. **B.** The mRNA transcript of S100P in highly invasive CL1-5 and less invasive CL1-0 cells. Sections of nontumorous and tumorous regions of lung cancer patients were co-stained by anti-S100P antibody and DAPI. S100P mRNA levels were determined by qRT-PCR. Data were shown as the mean±SD. *p < 0.05, or a significant difference between the two test groups.

Increased S100P expression occurred in the tumor region in a cohort of 24 lung cancer cDNA array library, compared to matching normal regions. S100P levels in the tumor region were elevated when compared to normal regions in 54.2% (13/24) of specimens (Table 1). There were significantly increased S100P protein levels in the tumor region of lung cancer specimens, compared to normal regions in the patients (*n* = 5), as determined by immunofluorescence staining (Figure 1A). Evaluated S100P mRNA transcript levels were also found in highly invasive lung cancer CL1-5 cells, when compared to isogenic less invasive CL1-0 cells (Figure 1B). These data demonstrate that S100P plays an oncogenic role in lung cancer.

### Knockdown of S100P alters cell morphology and tumor cell motility

**Figure 2:**
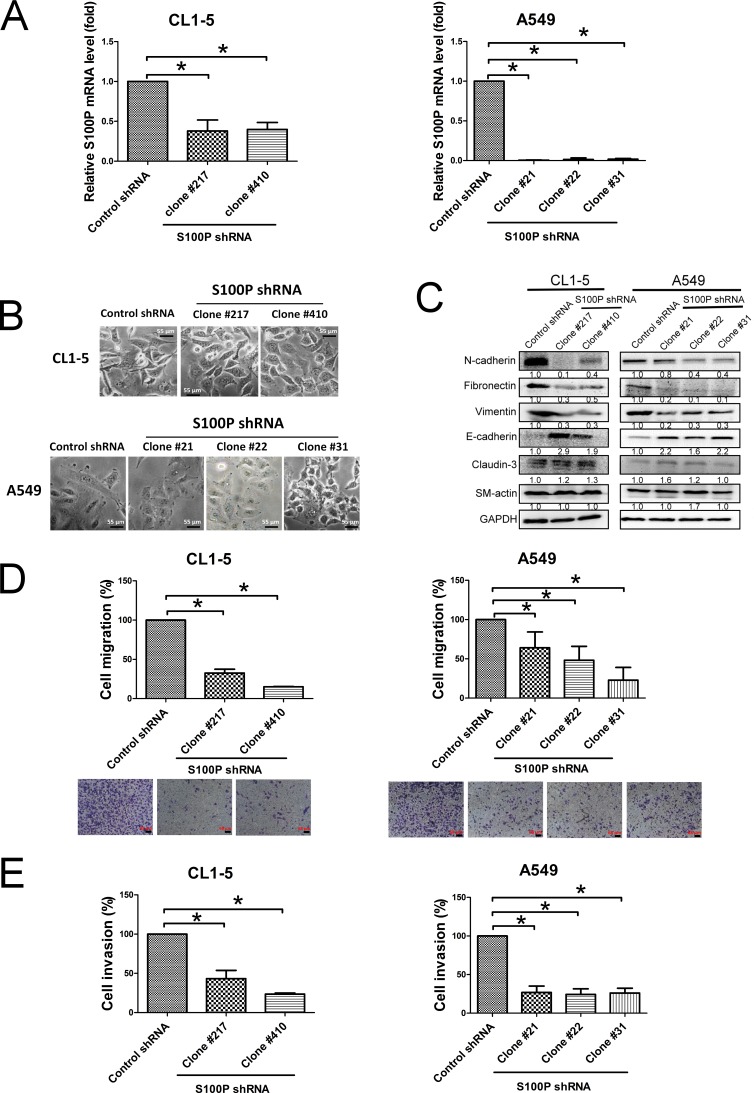
Loss of S100P protein caused MET and decreased cell migration and invasion in lung cancer cells **A.** Transfection of S100P shRNA plasmid decreased S100P. **B.** Morphologic imaging of S100P knockdown CL1-5 and A549 cells. **C.** Expression of the EMT markers as analyzed by immunoblot. S100P knockdown decreased cell migration **D.** and invasion **E.**. Protein levels were assessed by immunoblot. Migratory or invasive cells were stained by crystal violet or quantitated by a fluorescence dye. Data were shown as the mean±SD. *p < 0.05, or a significant difference between the two test groups.

To explore the role of S100P in lung cancer migration and invasion, endogenous S100P was stably inhibited by shRNA plasmid transfection in the CL1-5 and A549 lung cancer cell lines. The qRT-PCR of fractionated samples was used to measure the decreased expression of S100P in CL1-5 and A549 cells. Levels of S100P mRNA transcript (CL1-5 S100P KD clone 217 and 410, A549 S100P KD clone 21, 22 and 31) were reduced by approximately 70% and 90%, respectively, when compared to control plasmid transfected CL1-5 and A549 cells (CL1-5-AS2 and A549-AS2) (Figure 2A).

Silencing S100P resulted in MET supported by morphologic changes (from fibroblast-like shapes to epithelial features) (Figure 2B) and some protein expression (epithelial markers E-cadherin upregulation, and mesenchymal markers N-cadherin, fibronectin and vimentin downregulation) (Figure 2C). Knockdown of S100P also reduced metastatic features, including decreased cell migration, as determined by wound healing assay and transwell system (Suppl. Figure 1A; Figure 2D), and invasion in both CL1-5 and A549 cells (Figure 2E). However, inhibition of S100P did not affect CL1-5 and A549 cell proliferation (Suppl. Figure 1B). These data suggest that S100P plays an important role in cancer migration and invasion.

### S100P drove cell migration, invasion, and EMT in lung cancer

**Figure 3:**
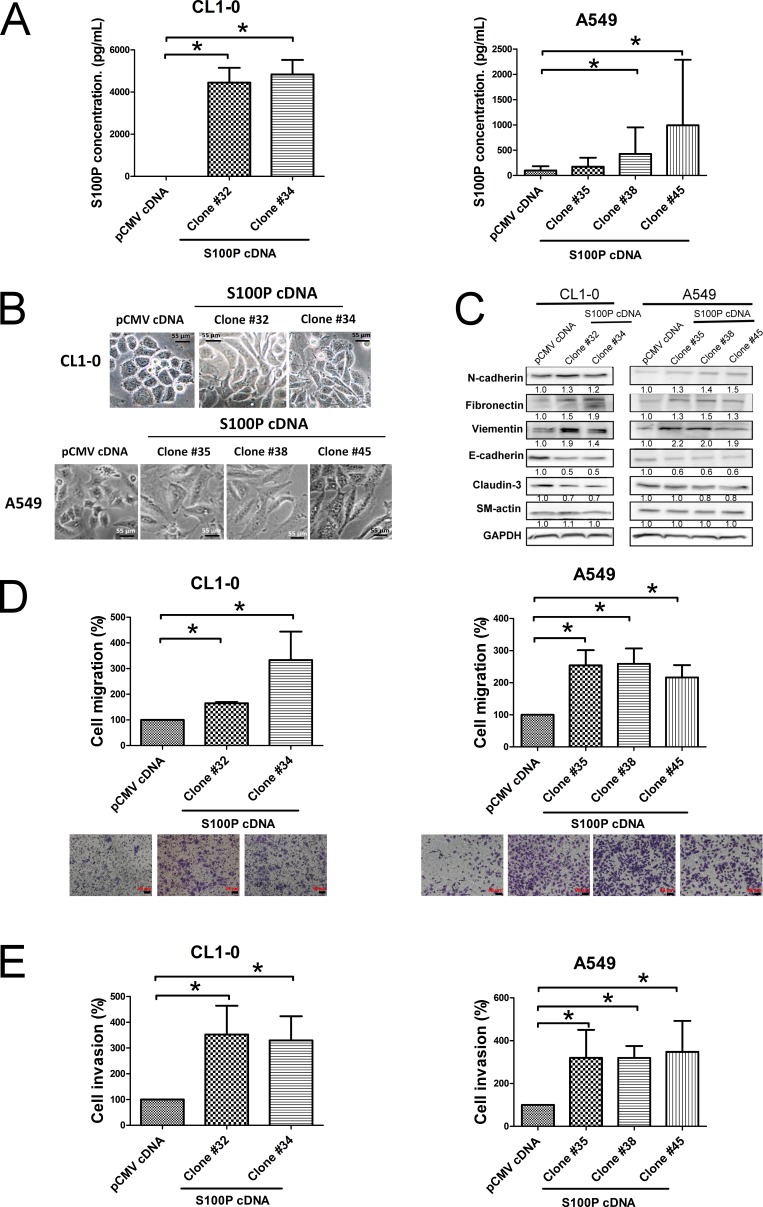
Overexpression of S100P protein caused EMT and increased cell migration and invasion **A.** The expression of S100P in CL1-0 and A549 cells. **B.** Morphologic change of S100P overexpressing CL1-0 and A549 cells. **C.** Expression of EMT markers. Ectopic expression of S100P increased cancer migration **D.** and invasion **E.**. The levels of various proteins were measured by immunoblot. Cell migration and invasion were determined as described above. Data were shown as the mean±SD. *p < 0.05, or a significant difference between the two test groups.

We further confirmed the role of S100P overexpression in lung cancer. After having transfected S100P cDNA into CL1-0 and A549 cells, ELISA analysis revealed that S100P cDNA transfection increased protein expression in CL1-0 and A549 cells, compared to pCMV plasmid cells (Figure 3A). The ectopic expression of S100P altered cell morphology from epithelial shapes to mesenchymal features (Figure 3B). The expression of epithelial marker E-cadherin decreased, whereas the mesenchymal markers N-cadherin, vimentin, and fibronection increased (Figure 3C). Moreover, metastatic characteristics, including cell migration, increased in both S100P overexpressing CL1-0 and A549 cells (Figures 3D and 3E; Suppl. Figure 2A). As seen in instances of S100P knockdown, overexpression of S100P did not affect cell proliferation in CL1-0 and A549 cells (Suppl. Figure 2B), which indicates that S100P increases lung cancer progression.

### S100P regulated lung cancer cell migration and EMT via ZEB1

**Figure 4:**
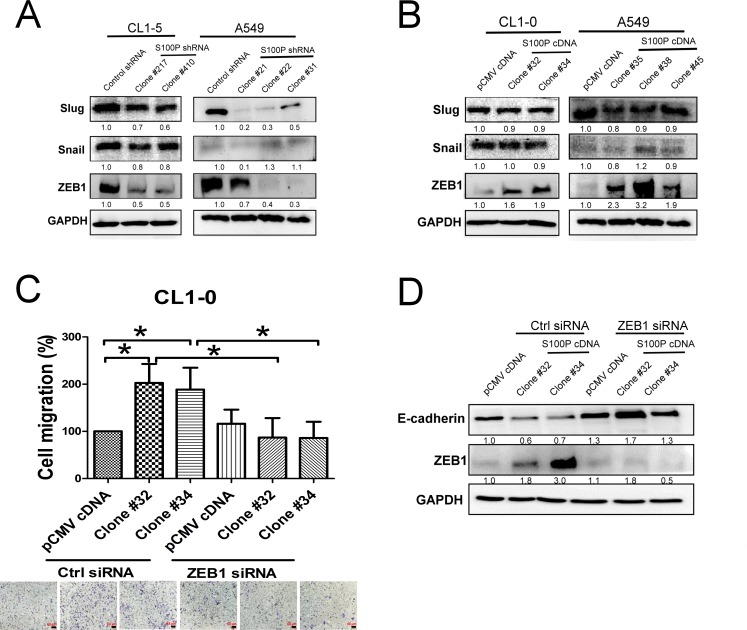
S100P-mediated EMT by ZEB1 The expression of EMT-related transcription factors in **A.** S100P-knockdown and **B.** overexpressing cells. **C.** Silencing of ZEB1 decreased cell migration in S100P overexpression cells. **D.** Inhibition of ZEB1 restored E-cadherin level in S100P overexpressing CL1-0. Ectopic expression of S100P CL1-0 cells was transfected with either control or ZEB1siRNA for 24 h. The cells were submitted to migration (trans-well assay) and immunoblot. Data were shown as the mean±SD. *p < 0.05, or a significant difference between the two test groups.

Transcriptional factors, including Snail, Slug and ZEB1 have been demonstrated to regulate EMT [15], we consequently assessed the effect of S100P in these types of protein expression. Knockdown of S100P decreased ZEB1 expression in both CL1-5 and A549 cells (Figure 4A). In contrast, overexpression of S100P increased ZEB1 levels in both CL1-0 and A549 cells. However, neither the knockdown nor the overexpression of S100P influenced Snail or Slug expressions in CL1-0 cells, although S100P inhibition did decrease Slug expression in A549 cells (Figures 4A and 4B).

To investigate the role of ZEB1 on S100P-mediated cancer migration and EMT, we inhibited ZEB1 by siRNA transfection, then assessed the expression of EMT markers and cell migration. Transfection of CL1-0 cells with ZEB1 decreased the mRNA transcript of ZEB1 (Suppl. Figure 3). Silencing ZEB1 decreased S100P-mediated cell migration (Figure 4C). The inhibition of ZEB1 also completely restored E-cadherin expression in S100P overexpressing CL1-0 cells (Figure 4D).

### S100P-knocked down lung cancer impaired the FAK/Src/Akt signaling pathway

**Figure 5:**
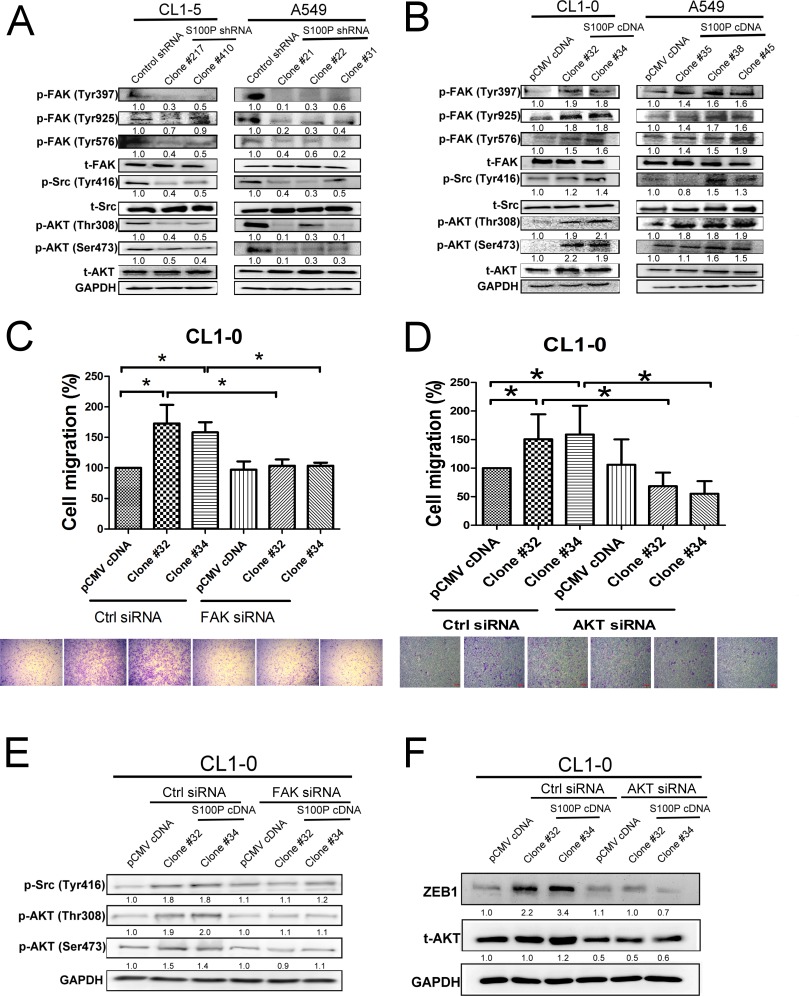
FAK/Src/AKT signaling is involved in S100P-mediated cancer **A.** Inhibition of S100P decreased the activation of FAK, Src, and AKT. **B.** Ectopic expression of S100P increased the phosphorylation of FAK, Src, and AKT. **C.** Inhibition of FAK by siRNA transfection prevented S100P-mediated cell migration. **D.** Knockdown of AKT by siRNA decreased S100P-mediated cell migration. **E.** Knockdown of FAK by siRNA reduced AKT and Src activation in S100P overexpressing CL1-0 cells. **F.** Transfection of AKT siRNA decreased ZEB1 upregulation in S100P overexpressing CL1-0 cells. The cells were submitted to migration (transwell assay) and immunoblot. The expressions of various proteins were assessed by immunoblot. Data were shown as the mean±SD. *p < 0.05 or significant difference between control and test groups.

Consistent with a role in modulating cell movement, the S100P knockdown CL1-5 and A549 cells exhibited decreased FAK (Tyr 397, 925, and 576), Src (Tyr 416), and AKT (Thr 308 and Ser 473) phosphorylation, without any obvious alteration in their unphosphorylated forms. In contrast, ectopic expression of S100P protein displayed increased FAK, Src, and AKT activation in CL1-0 and A549 cells (Figure 5B).

To discern whether FAK and AKT activation initiated S100P-mediated cancer migration, FAK and AKT siRNA transfection were assayed on cancer migration in S100P overexpressing CL1-0 cells. An inductive effect on cell migration by S100P overexpression was decreased by either FAK or AKT siRNA transfection (Figure 5C and 5D). Knockdown of FAK siRNA also decreased the phosphorylation of Src and AKT in S100P overexpressing CL1-0 cells (Figure 5E). FAK inhibitor also prevented cell migration and activation of Src and AKT (Suppl. Figure 4A and 4B). Furthermore, AKT knockdown prevented S100P-mediated ZEB1 upregulation in CL1-0 cells (Figure 5F), suggesting that the FAK/AKT/ZEB1 axis plays a role in S100P-mediated cancer EMT and migration.

### S100P increased lung cancer migration by integrin α7, but not RAGE

Previous studies have shown that cell surface molecules, such as RAGE (receptor for advanced glycation end products) and integrins, are receptors for S100 family proteins [7, 8]. We investigated the possible role of RAGE and integrins on S100P-mediated lung cancer progression. RAGE inhibitor did not affect S100P-mediated cell migration, indicating that RAGE was not a mediator of S100P-induced cancer progression (Suppl. Figure 5).

**Figure 6:**
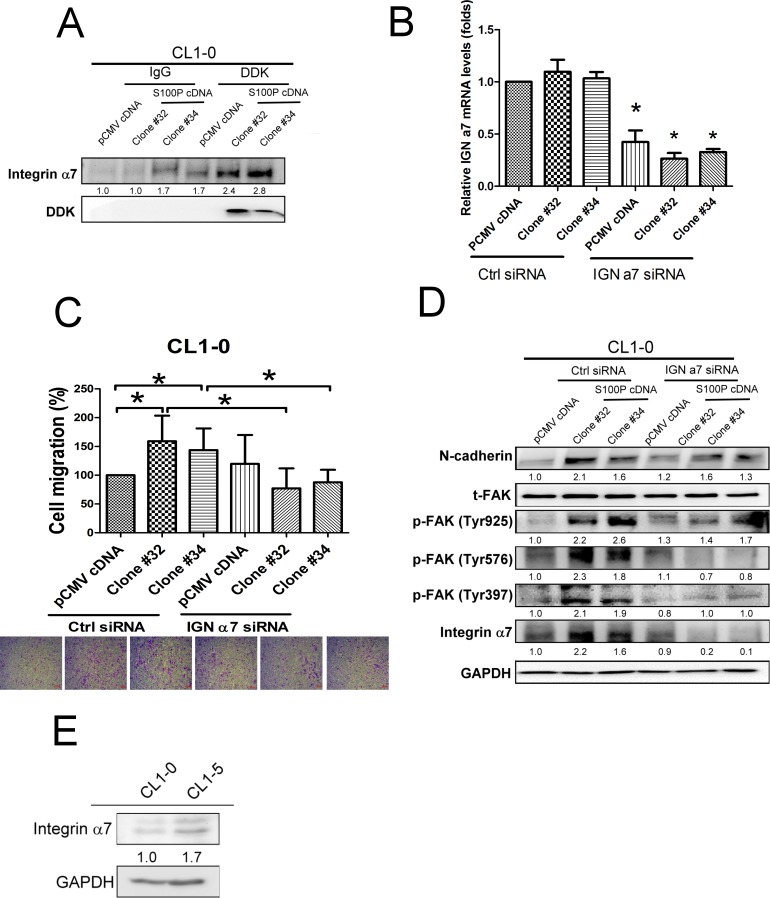
Integrin α7 was involved in S100P-mediated cancer progression **A.** The interaction of S100P with integrin α7. **B.** The efficacy of integrin α7 siRNA transfection. **C.** Knockdown of integrin α7 decreased S100P-mediated cell migration. **D.** Inhibition of integrin α7 reduced N-cadherin upregulation and FAK in S100P overexpressing CL1-0 cells. **E.** The expression of integrin α7 in CL1-0 and CL1-5 cells. Levels of integrin α7 were determined using anti-integrin α7 antibody. The cells were submitted to migration (transwell assay) and immunoblot. The expressions of various proteins were assessed by immunoblot. Data were shown as mean±SD. *p < 0.05 or significant difference between control and test groups.

To investigate the candidate of S100P binding receptor, we pulled down S100P from the cell membrane of S100P overexpressing CL1-0 cells, then determined the protein prolife by nanoUPLC-MS/MS. Mass spectrometry analysis shows integrin α7 abounds in S100P protein precipitant of CL1-0 cell membranes. Coimmunoprecipitation data revealed that S100P was bound to integrin α7 (Figure 6A). The role of integrin α7 was further assessed by siRNA-based inhibition. Transfection of control and S100P overexpressing CL1-0 cells with integrin α7 siRNA decreased the expression of integrin α7 by approximately 67% (Figure 6B). Knockdown integrin α7 by siRNA transfection decreased cell migration in S100P overexpressing CL1-0 cells (Figure 6C). Inhibition of integrin α7 also prevented the activation of FAK and N-cadherin upregulation induced by S100P overexpressing CL1-0 cells (Figure 6D). Evaluated integrin α7 protein levels were also found in CL1-5 cells, in comparison with CL1-0 cells (Figure 6E). These data suggest that integrin α7 appears to be the binding receptor for S100P, and subsequently mediated the upregulation of cell mobility by activating FAK/AKT signaling.

### Knockdown of S100P decreased lung cancer metastasis *in vivo*

**Figure 7:**
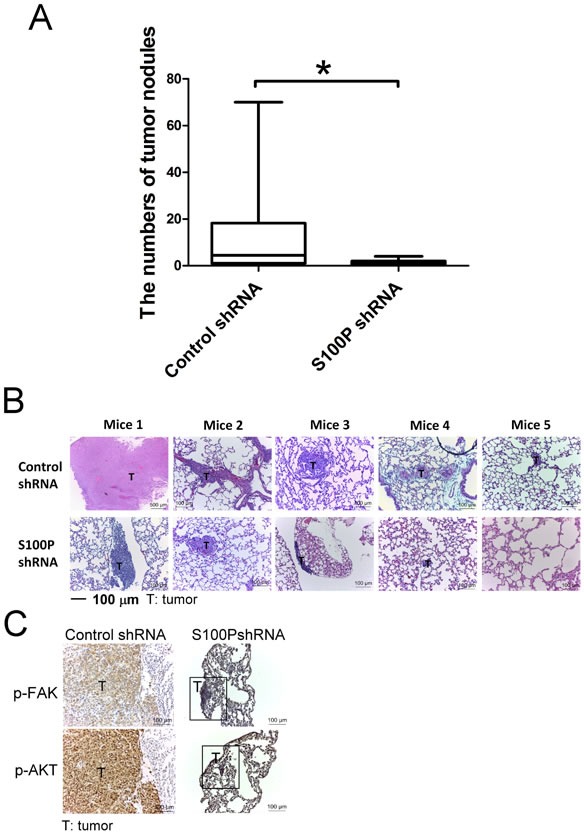
Inhibition of S100P decreased lung cancer metastasis *in vivo* **A.** The lung tumor nodules of mice. **B.** H&E staining of lung tumor sections. **C.** The phosphorylation of FAK and AKT in lung tumors of mice. *p < 0.05 or significant difference in control shRNA plasmid vs. S100P shRNA plasmid, as analyzed by Student's *t* test.

To investigate the influence of S100P in lung cancer metastasis, S100P-knockdown CL1-5 was transplanted into mice by tail vein injection. Inhibition of S100P decreased the metastatic ability of CL1-5 in mice (Figure 7A). As determined by H&E staining, the control plasmid-transfected CL1-5 cell tumors were larger and more invasive than those of S100P-knockdown cells (Figure 7B). In addition, IHC results also revealed that knockdown S100P decreased the phosphorylation of FAK and AKT in the S100P knockdown CL1-5 tumors of mice, compared to the tumors of control plasmid transfected CL1-5 cells (Figure 7C).

## DISCUSSION

Increased S100P levels have been reported in liver, skin, endometrial, colon, and pancreatic cancers, and have been indirectly implicated in promoting carcinogenesis and cancer metastasis in animal models [5, 6, 16-18]. However, the exact molecular mechanism involved in the promotion of lung cancer cell progression remains unclear. This study demonstrates that S100P is highly expressed in the tumor region of lung cancer tissues and in the highly invasive lung cancer cell CL1-5, but is weakly expressed in normal regions of lung tissue and the less invasive CL1-0 cell line. Knockdown of S100P in CL1-5 and A549 cells leads to MET, together with decreased cell migration and invasion ability. Conversely, overexpression of S100P in CL1-0 and A549 cells causes EMT and increased cancer movement and invasion potential. Moreover, decreased S100P expression reduces the metastatic ability of CL1-5 in mice. These results reveal that S100P may be an important regulator of lung cancer progression.

The occurrence and progression of cancer metastasis are complicated. First, tumor cells disassociate from the primary tumor site by losing cell-cell connection and switching to a mesenchymal phenotype, resulting in increased movement and invasiveness [19, 20]. The change of epithelial features to a mesenchymal phenotype is accomplished by the downregulation of tight junctions and the upregulation of focal adhering proteins [21]. The conversion of an epithelial phenotype to mesenchymal properties is strongly related to cancer metastasis, resulting in poor prognoses for lung cancer patients [22, 23].

This study demonstrates that overexpression of S100P in CL1-0 or A549 cells increases the ability for migration, invasion, and EMT. Conversely, in metastatic CL1-5 or A549 cells that express high levels of endogenous S100P, knockdown of S100P decreases migration and invasion abilities, causing MET. Furthermore, inhibition of S100P in CL1-5 decreases the metastatic recurrence of CL1-5 in mice. More importantly, the tumor regions of specimens from lung cancer patients express higher amounts of S100P when compared to matching normal regions. Results from cell-based studies, mouse models, and clinical patient specimens suggest that S100P is a critical mediator contributing to the development of lung cancer.

ZEB1, zinc finger transcription factor, is essential for regulating EMT and cancer metastasis [24, 25]. ZEB family proteins have been indicated to regulate cell cycle progression, apoptotic cell death and senescence, and to induce the epithelial dedifferentiation of cancer-initiating cells, leading to carcinogenesis and cancer progression [26]. Diffuse expression of ZEB1 is associated with poor survival rates in lung cancer patients [27]. AKT has been reported to regulate ZEB1 by inhibiting miR-200 and activating the c-myc pathway [28, 29]. Results of the present study also reveal that S100P activates FAK and AKT signaling. Inhibition of FAK decreases AKT phosphorylation induced by S100P, suggesting that FAK is the upstream regulator of AKT.

In addition, knockdown of AKT prevents ZEB1 upregulation and EMT, showing that AKT activation is responsible for ZEB1 upregulation. Silencing of ZEB1 by siRNA transfection also decreases the switch of EMT induced by S100P overexpression. Such findings support the phenotypic transition of lung cancer by S100P mediated FAK-mediated activation of AKT and subsequent AKT-activated ZEB1 gene expression, thereby contributing to cell migration.

Integrin family proteins, the family of heterodimeric transmembrane adhesion receptors, have been implicated in the pathogenesis of various malignances, including lung cancer [11, 30]. The role of integrin α7 in cancer is controversial. It has been reported to prevent cell proliferation and induce cell death by binding to tissue inhibitor of metalloproteinase 3 and high temperature requirement A2 in prostate cancer [31, 32]. However, recent studies indicate that its expression is higher in metastatic hepatocellular carcinoma specimens than in non-metastatic HCC specimens, whereas its inhibition by meta-iodobenzylguanidine decreases migration of liver cancer cells [33]. Cigarette smoke extract (CSE) and CCN family member 1 increase lung epithelial cells to express matrix metalloproteinase 1 in an integrin α7-dependent manner, which destroys basement membrane and stromal matrix, resulting in cancer metastasis [34, 35]. Furthermore, transfection of integrin α7 induces cell motility in non-motile HEK293 cells [36]. This study is the first to demonstrate that S100P interacts with integrin α7, which in turn increases the activation of FAK and AKT. Knockdown of integrin α7 prevents cell migration, EMT (N-cadherin upregulation), and FAK activation in S100P overexpressing cancer cells, suggesting that S100P promotes cancer progression by binding integrin α7 and the FAK/AKT signaling pathway.

In conclusion, S100P interacts with integrin α7 to regulate lung cancer cell migration and invasion via the FAK/AKT-ZEB1 signaling pathway. This suggests that S100P plays a pivotal role in integrin-mediated cancer progression, and that the inhibition of S100P may be a promising target for diagnostic and therapeutic interventions against metastatic events.

## MATERIALS AND METHODS

### Antibodies and reagents

Primary antibodies against phospho-Tyr 397 FAK, total FAK, phospho-Ser 473 Akt, total Akt, phospho-Tyr 416 Src, total Src, vimentin, snail, slug, and ZEB1 were purchased from Cell Signaling Technology (Boston, MA, USA). Antibodies against anti-human S100P and claudin-3 (Abcam, San Francisco, CA, USA), SM-actin (Sigma-Aldrich, St. Louis, MO), Fibronectin, N-cadherin and E-cadherin (BD Biosciences, Bedford, MA) were obtained from their respective companies. Primary antibodies against GAPDH, FAK, and AKT inhibitors were purchased from Millipore (Billerica, MA). S100P ELISA kit was obtained from CircuLex (Woburn, MA).

### Cell lines and tumor samples

Less invasive (CL1-0) and highly invasive (CL1-5) human lung adenocarcinoma cell lines were provided by Dr. Pan-Chyr Yang (Department of Internal Medicine, National Taiwan University Hospital) [37, 38]. These were cultured in RPMI 1640 supplemented with 10% fetal bovine serum (FBS) and 1% penicillin-streptomycin (Gibco BRL, Life Technologies). Human lung cancer cell line A549 was obtained from the American Type Culture Collection (number CCL-185) and cultured in F-12K Nutrient Mixture Kaigh's modified with 10% fetal bovine serum (FBS) and 1% penicillin-streptomycin (Gibco BRL, Life Technologies).

### S100P knockdown and overexpression, stable clone generation, and siRNA transfection

Knockdown of S100P in CL1-5 and A549 was performed using a lentiviral expression system obtained from the National RNAi Core Facility (Taipei, Taiwan). The lentiviruses were produced by co-transfecting HEK293T with pLKO-AS2, pLKO-AS3-S100P and two packaging plasmids (pCMVVDR8.91 and pMD.G). Stable clones were established by puromycin selection. ON-TARGET plus SMARTpool siRNA (Thermo Fisher Scientific, Waltham, USA) transfection was used for ZEB1, AKT1 and FAK knockdown

Quantitative reverse transcriptional polymerase chain reaction (qRT-PCR) was used to assess the efficacy of the S100P shRNA plasmid and various siRNA transfection. Overexpression of S100P in CL1-0 and A549 was achieved by transfecting S100P cDNA (Origene Technologies, Rockville, MD, USA). Stable clones were established by G418 selection.

### Quantitative reverse transcriptional PCR (qRT-PCR)

Cells were harvested using TRIzol reagent (Invitrogen, Carlsbad, CA), followed by RNA extraction according to the manufacturer's instructions. Equal amounts of RNA were used to synthesize first-strand cDNA. RT-PCR was performed using SYBR Green on a StepOnePlus Real-Time PCR System (Applied Biosystems, Foster City, CA). Each PCR reaction mixture contained 200 nM of each primer, 5 μL 2×SYBR Green PCR Master Mix (Applied Biosystems, Foster City, CA), 5 μl cDNA, and RNase-free water, for a total volume of 10 μL.

The PCR reaction was conducted with a denaturation step at 95°C for 20 sec, 40 cycles at 95°C for 3 sec, and 60°C for 30 sec. All PCRs were performed in triplicate and normalized to internal control GAPDH mRNA. Relative expressions were presented using the 2^−ΔΔCT^ method. S100P levels in Lung Cancer cDNA Array I (Origene) were also measured by PCR. The primer sets used were S100P: 5′- TACCAGGCTTCCTGCAGAGT-3′ and 5′- CACGTCTGCCTGTCACAAGT-3′; RAGE: 5′- CAGTGTGGCTCGTGTCCTT-3′ and 5′- CAGTGTGGCTCGTGTCCTT-3′; and GAPDH: 5′- GAGTCAACGGATTTGGTCGT-3′ and 5′- TTGATTTTGGAGGGATCTCG-3′.

### Wound healing, migration, and invasion assay

Cell migration and invasion was analyzed *in vitro* using CHEMICON QCM 24 well migration or invasion assay kits (Millipore, Bedford, MA). Briefly, cells were seeded into each insert (pore side: 8 μm) of the 24-well plate and incubated at 37°C for 24 h. After using cotton swabs to remove the cells on the upper side of the insert membrane, the cells on the bottom side of the insert membrane were fixed with methanol for 15 min, followed by staining with crystal violet for 60 min. After washing with distilled water, the invaded cells on the membrane were counted in three random fields of view per trans-well. The images were captured via microscope. Alternatively, the migratory and invading cells was quantified by fluorescence dye staining, and the OD value read by a fluorescence plate reader at excitation/emission wavelengths of 485/530 nm. The migration of A549 cells was also assessed by Scratch wound-healing assay.

### Immunoblot/Coimmunoprecipitation (Co-IP)

The cells were lysed on ice for 30 min in M-PER solution. Cell lysate was centrifuged at 12,000 rpm for 15 min and the supernatant fraction was collected for immunoblot. Equivalent amounts of protein were resolved by SDS-PAGE (10-12%) and transferred to PVDF membranes. After blocking in 5% non-fat dry milk in Tris-buffered saline, the membranes were incubated overnight with the desired primary antibody. The membranes were then treated with the appropriate peroxidase-conjugated secondary antibody and the immuno-reactive proteins were detected by an enhanced chemi-luminescence kit (Millipore), according to the manufacturer's instructions. To quantify the immunoblot images on unsaturated bands, densitometric analysis was performed using AlphaEaseFC (Alpha Innotech).

For Co-IP, cell lysates (200 μg of total protein) were incubated with 2 μg of isotype or anti-DDK antibodies (Origene) overnight, and then with 10 μL protein A-agarose beads (Millipore, Bedford, MA) for 2 h at 4°C. DDK and intergrin α7 were detected by incubating the blots with specific antibodies.

### Protein identification by nanoUPLC-MS/MS

Cell membrane protein was extracted by the Novagen proteoextract Transmembrane protein Extraction Kit (Millipore). Protein solution 10 μL (∼10 μg) was mixed well with 100 μL acetone and centrifuged at 13,000 rpm for 10 min. After centrifugation, the supernatant was discarded, and the protein residues kept and dried by evaporation. Protein residues were then re-dissolved in 18 μL 25 mM ammonium bicarbonate aqueous solution. Then 2 μL sequence-grade trypsin (0.1 μg /μL, Promega, Madison, WI, USA) was added and protein solutions were digested at 37°C for 16 h. After trypsin digestion, 2 μL tryptic peptide solution was injected into the nano LC system and detected by LTQ Orbitrap.

The LC-MS/MS conditions were LTQ Orbitrap Discovery hybrid FTMS (Fourier Transform Mass Spectrometer) (Thermo Fisher Scientific, Inc. Bremen, Germany) at 30000 resolution, with a nanospray source in the positive ion mode. The nano UPLC system (nanoACQUITY UPLC) was manufactured by Waters (Milford, MA, USA). The desalting column (Symmetry C18, 5 μm, 180 μm x 20 mm) and analytical column (BEH C18, 1.7 μm, 75 μm x 150 mm) were also purchased from Waters. Mobile phase A was the aqueous solution of 0.1 % formic acid, and mobile phase B was the organic solvent (100% ACN containing 0.1% formic acid).

After desalting by 0.1% formic acid for 3 min at a flow rate of 5 μL/min, analysts were separated at a flow rate of 300 nL/min by a 15 cm UPLC column. The gradient conditions were t: 0-1 min, hold B: 1%; t: 1-20 min, B 1-45%; t: 20-30 min, B 45-85%; t: 30-35 min, B 85%; t: 35-45 min, B 85-1%; and t: 45-60 min, B 1%.

Peptide elute from the column was directed to the nano-spray source and the MS was operated in the positive ion mode. The MS/MS was acquired with a mass spectrometer operated in the data-dependent mode. Individual raw data files were processed with Mascot Distiller (Version 2.2, Matrix Science Inc., Boston, MA) software and then uploaded to the in-house Mascot server (Version 2.2, Matrix Science Inc., Boston, MA) for protein identification.

### Immunofluorescence

Noncancerous and cancerous lung tissue specimens from human lung cancer patients were embedded in OCT and frozen in liquid nitrogen. Sections (5 μm) were fixed with 4% paraformaldehyde, permeabilized with Triton (0.25%), and stained with S100P antibody (1:100, Abcam, UK). After washing with PBS containing 0.1% Tween-20 (PBST), slides were incubated with Dylight 488-conjugated secondary antibodies (1:500, Rockland, Gilbertsville, PA), with or without DAPI, for 1 h at room temperature. The data were analyzed with a confocal laser-scanning microscope (LSM700, Zeiss Germany).

### Metastasis of cancer *in vivo*

Control plasmid transfected CL1-5 and S100P-knockdown CL1-5 (clone 217) were transplanted into nude mice by tail vein injection. Animals were sacrificed on weeks 12 and the number of tumor nodules was recorded for analysis of lung cancer incidence. All immunohistochemical reactions were performed on 8-μm-thick paraffin sections. In brief, the sections were deparaffinized in xylene and rehydrated, then incubated in target retrieval solution (DAKO) in an autoclave for 8 min to retrieve the antigens. Endogenous peroxidase activity was blocked by 10 minutes of incubation with a 3% solution of H_2_O_2_. The expressions of phospho-FAK, and -AKT antigen were demonstrated using mouse/rabbit anti human-p-FAK and p-AKT (dilution 1:100) antibody. The sections were incubated with the primary antibodies overnight at 4°C. The studied antigens were then visualized using biotinylated antibodies and streptavidin, conjugated with horseradish peroxidase. Diaminobenzidine Dako Cytomation (Glostrup, Denmark) served as the substrate, and all of the sections were counterstained with hematoxylin.

### Statistical analysis

A two-tailed Student's *t*-test was used when comparing the means of two groups. Statistical significance was set at *p* < 0.05.

## SUPPLEMENTARY MATERIAL FIGURES AND TABLE


