# Supplementary material for: S100P interacts with integrin α7 and increases cancer cell migration and invasion in lung cancer
Source: Oncotarget. 2015 Jul 21;6(30):29585–98. doi: 10.18632/oncotarget.4987 (PMC4745748; doi:10.18632/oncotarget.4987)
Supplement: Supplementary file 1 [file oncotarget-06-29585-s001.pdf]

# S100P interacts with integrin α7 and increases cancer cell migration and invasion in lung cancer

**Supplementary Material**

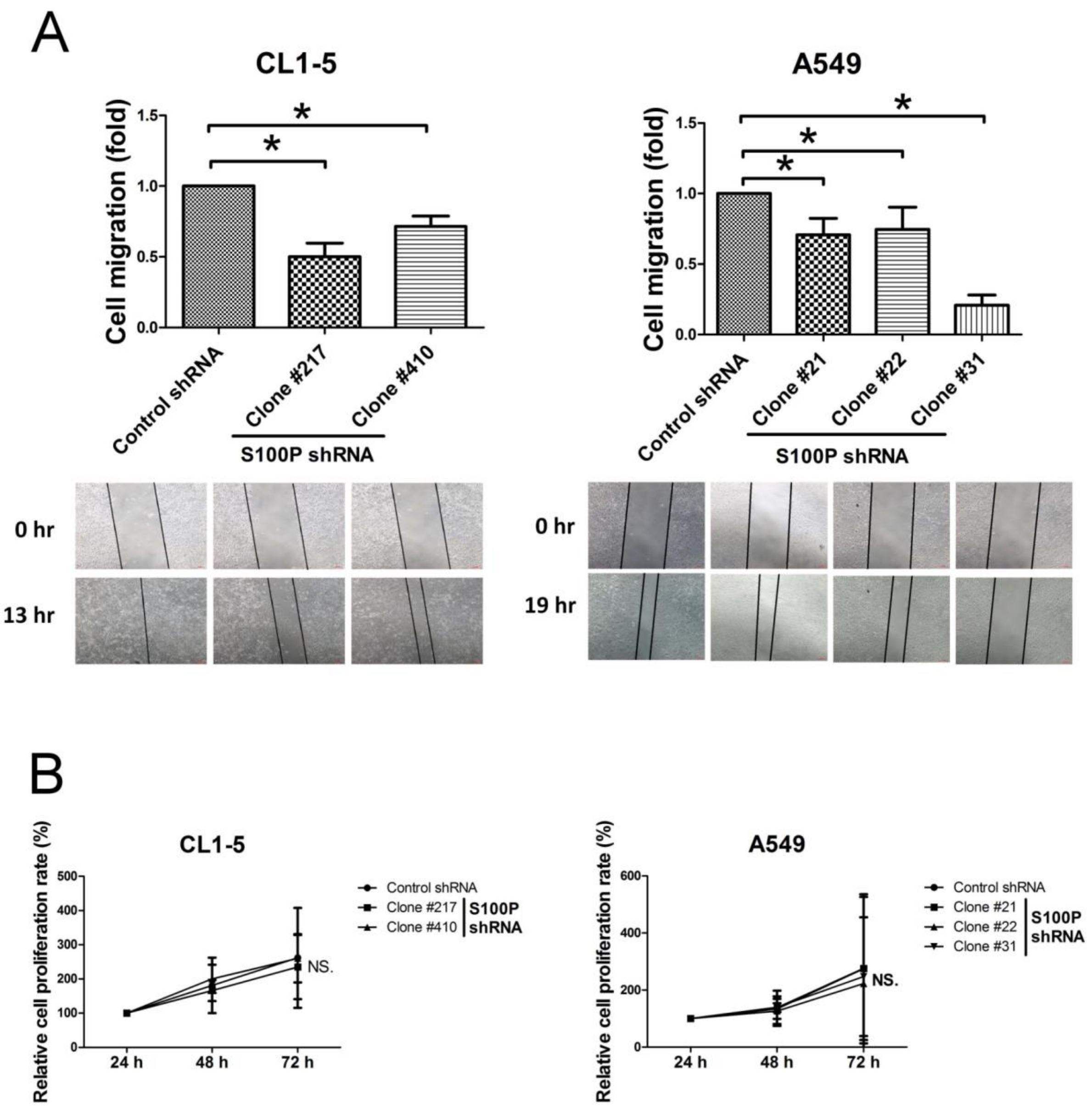


**Figure 1. Inhibition of S100P reduced cell migration.** Knockdown of S100P **(A)** decreased cell migration, but **(B)** did not affect cell proliferation in CL1-5 and A549 cells, as assessed by wound-healing assay or by WST-1 for indicated times. Data were as the mean±SD. *$p$<0.05 or significant difference between control and test groups; ns, non-significant

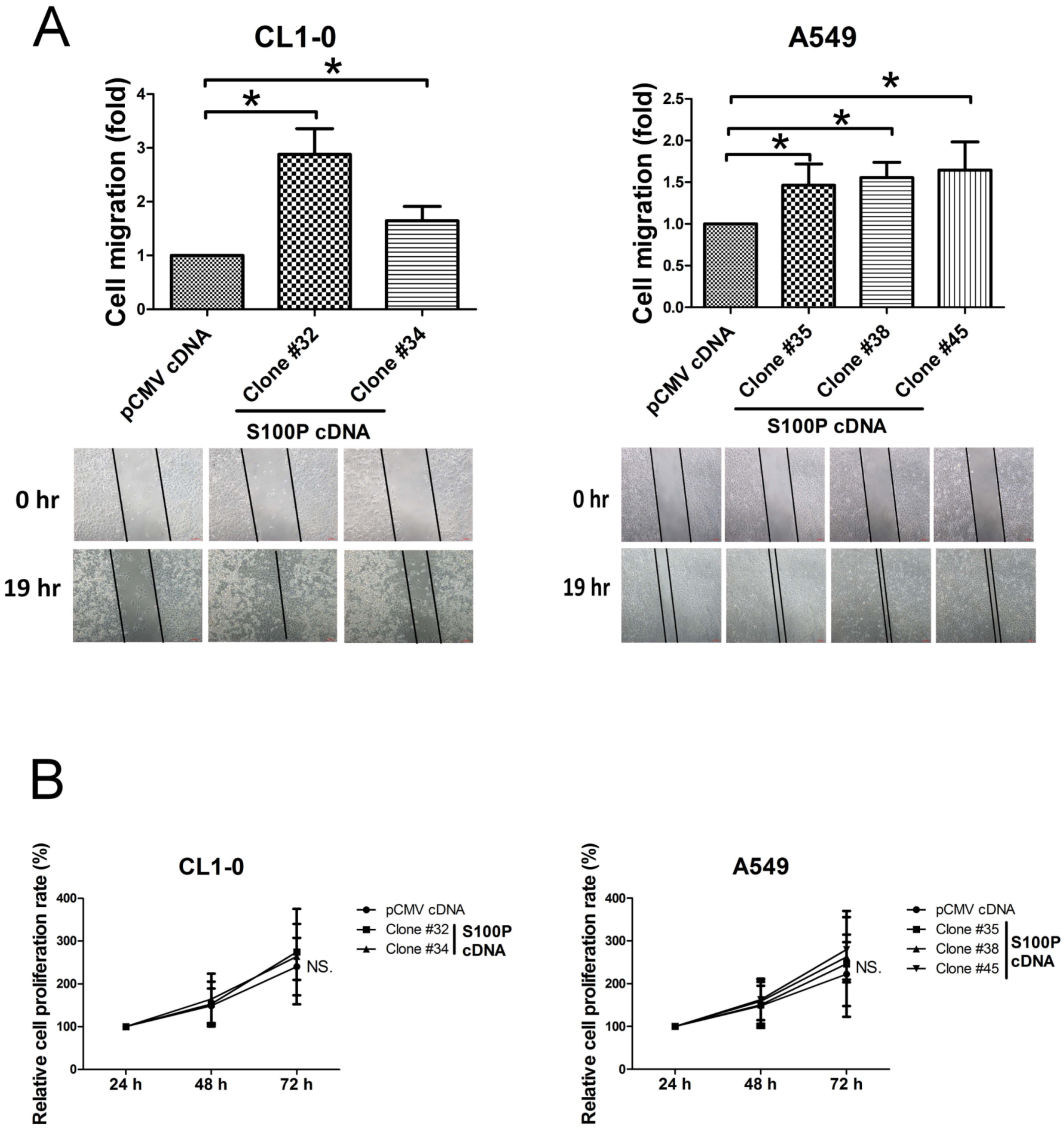


**Figure 2. Overexpression of S100P increased cell migration.** Elevated S100P **(A)** enhanced cell migration, but **(B)** did not affect cell proliferation in CL1-0 and A549 cells, as assessed by wound healing assay or by WST-1 for indicated times. Data were provided as the mean±SD. *$p$<0.05 or significant difference between control and test groups; ns, non-significant

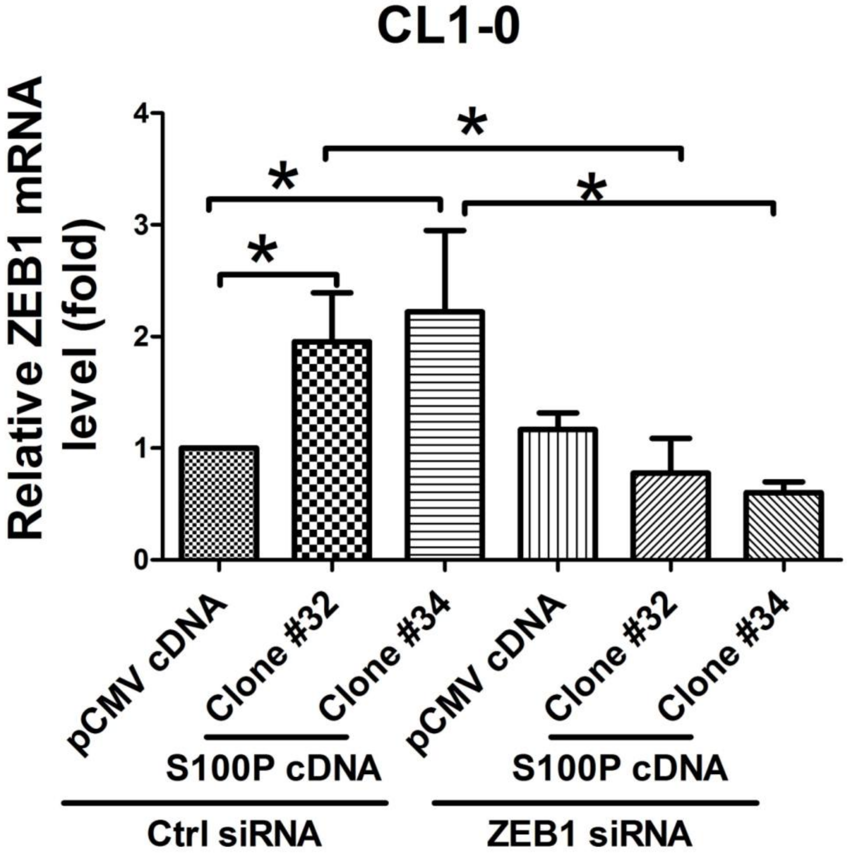


**Figure 3. The expression of ZEB1.** S100P overexpressing CL1-0 cells were transfected with either control or ZEB1 siRNA. The mRNA level of ZEB1 was determined by qRT-PCR 24 h after transfection. Data were as the mean±SD. *$p$<0.05, or a significant difference between the two test groups

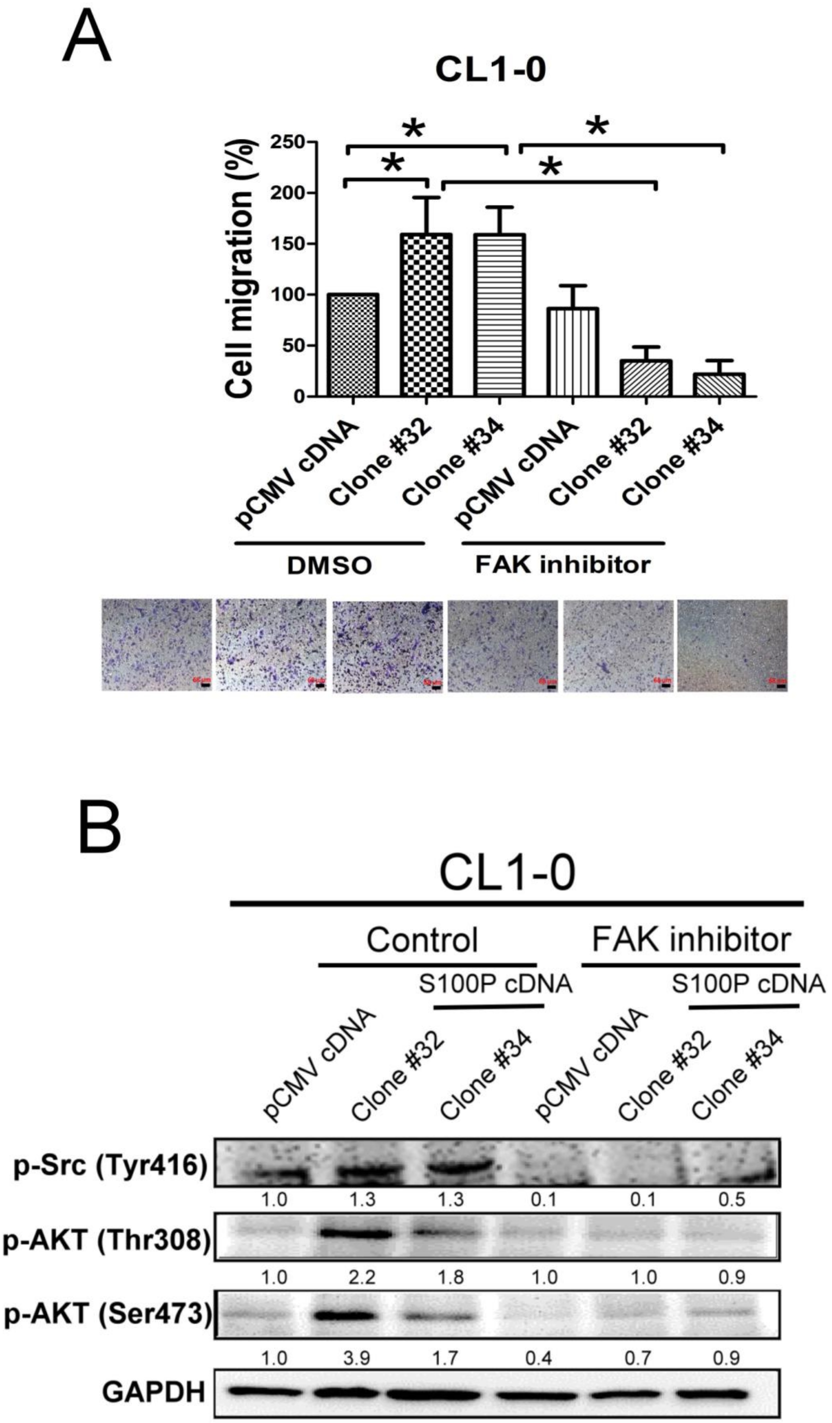


**Figure 4. The role of FAK on cell migration and activation of Src and AKT.** S100P overexpressing CL1-0 cells were treated with FAK inhibitors (20 $\mu$M) and cell migration was assessed by the transwell system. The levels of various protein were assessed by immunoblot. Data were as mean±SD. *$p<0.05$, or a significant difference between the two test groups.

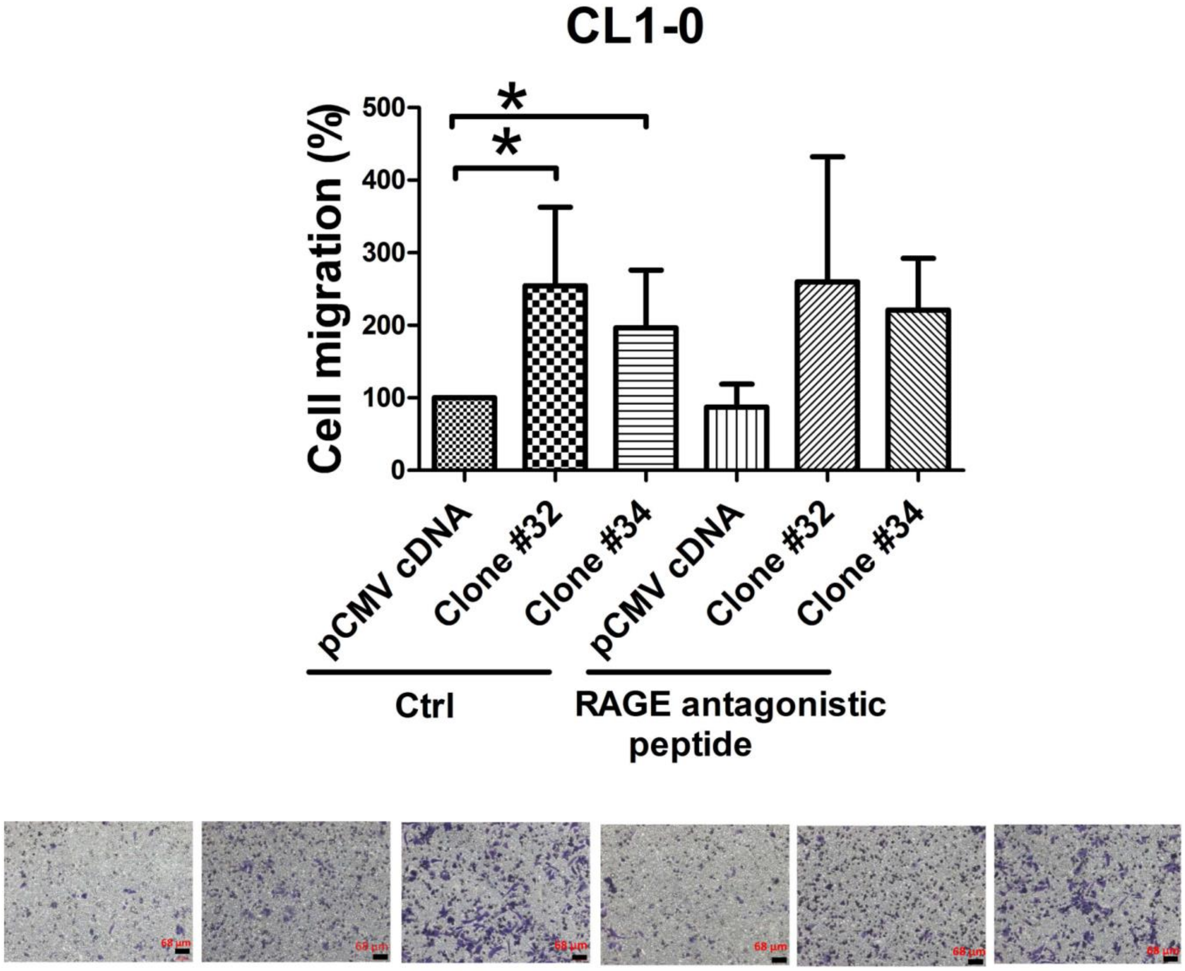


**Figure 5. RAGE inhibition did not affect the S100P induced cell migration.** S100P overexpressing CL1-0 cells were treated with RAGE inhibitors and cell migration was assessed by the transwell system. Data were as mean±SD. *$p$<0.05, or a significant difference between the two test groups.

**Table 1.** The mRNA transcript of S100P in non-tumor and tumor region in a cDNA array of lung cancer patients

| | | S100P | | | | | |
|---|---|---|---|---|---|---|---|
| | | **High expression in tumor region** | | **Low expression in tumor region** | | **No difference in tumor region** | |
| | N | n | % | n | % | n | % |
| **Tumor** | 24 | 13 | 54.2% | 7 | 29.2% | 4 | 16.6% |
| **Gender** | 24 | | | | | | |
| Male | 16 | 10 | 62.5% | 4 | 25.0% | 2 | 12.5% |
| Female | 8 | 3 | 37.5% | 3 | 37.5% | 2 | 25.0% |
